# Balanced Leader Distribution Algorithm in Kubernetes Clusters

**DOI:** 10.3390/s21030869

**Published:** 2021-01-28

**Authors:** Nguyen Dinh Nguyen, Taehong Kim

**Affiliations:** School of Information and Communication Engineering, Chungbuk National University, Cheongju, Chungbuk 28644, Korea; nguyennd@cbnu.ac.kr

**Keywords:** containers, Kubernetes, leader election, load balancing, stateful

## Abstract

Container-based virtualization is becoming a de facto way to build and deploy applications because of its simplicity and convenience. Kubernetes is a well-known open-source project that provides an orchestration platform for containerized applications. An application in Kubernetes can contain multiple replicas to achieve high scalability and availability. Stateless applications have no requirement for persistent storage; however, stateful applications require persistent storage for each replica. Therefore, stateful applications usually require a strong consistency of data among replicas. To achieve this, the application often relies on a leader, which is responsible for maintaining consistency and coordinating tasks among replicas. This leads to a problem that the leader often has heavy loads due to its inherent design. In a Kubernetes cluster, having the leaders of multiple applications concentrated in a specific node may become a bottleneck within the system. In this paper, we propose a leader election algorithm that overcomes the bottleneck problem by evenly distributing the leaders throughout nodes in the cluster. We also conduct experiments to prove the correctness and effectiveness of our leader election algorithm compared with a default algorithm in Kubernetes.

## 1. Introduction

Recently, container-based virtualization has emerged as a key technology to deploy applications in Cloud computing [1]. Unlike traditional virtual machines [2], a container runs at the software level within a host machine and shares the kernel with the host operating system [3]. It consumes fewer resources than the traditional virtualization method because it does not consist of an entire operating system; only the application and its dependencies are bundled into a single package. These features make containers more efficient in the deployment and scalability of applications.

In a large-scale system, it is important to have an orchestration platform to manage the container deployment. Kubernetes [4] is the most popular orchestration platform for container-based applications. It provides several powerful functions, such as automated application deployment, resource management, scaling, and load balancing. In a Kubernetes cluster, the application, which contains several replicas, is generally categorized as either stateless or stateful [5]. A stateless application has no persistent storage associated with it, whereas a stateful application requires a persistent datastore. This means that each replica in the stateful application should have its own persistent datastore. Therefore, it is important to maintain consistency among these distributed data stores of the stateful application. This consistency problem can be handled using a leader-based consistency maintenance mechanism in which an elected leader is responsible for maintaining consistency and coordinating tasks among replicas. Kubernetes provides a leader election algorithm, which is implemented by leveraging existing components in Kubernetes, to facilitate the process of using leader election in a Kubernetes cluster [6]. In the leader-based mechanism, the leader solely handles all the data update requests; therefore, the leader replica consumes a load that is heavier than that consumed by other replicas owing to the inherent design of the mechanism.

In this paper, we target a scenario in which the Kubernetes cluster acts as a Fog computing infrastructure, and several stateful applications that use a leader-based mechanism are deployed in the infrastructure. If the leaders of these applications are concentrated on a specific node, it can cause a problem wherein user requests also concentrate on a single node, possibly resulting in a bottleneck within the system. Please note that the preliminary version of this paper [7] proved that the default leader algorithm in Kubernetes does not consider such leader concentration problem that results in a significant performance reduction. Therefore, to improve the performance of the system, it is highly required to balance the number of leaders among nodes in the cluster. In this paper, we propose a balanced leader distribution (BLD) algorithm for the leader election process of stateful applications in Kubernetes clusters. The BLD algorithm improves the default leader election algorithm in Kubernetes by evenly distributing leaders throughout the nodes, thereby balancing the workload among nodes in the cluster. Through experimental evaluations, we verified the correctness and effectiveness of the BLD algorithm in Kubernetes clusters. Consequently, the main contributions of the BLD algorithm can be summarized as follows:

- It facilitates the use of the leader election process, so users can easily deploy the leader election for application in the Kubernetes cluster.
- It balances the number of leaders throughout all the nodes in the cluster, so the system performance can be improved.

The remainder of this paper is organized as follows. Section 2 presents related work. Section 3 provides an overview of the Kubernetes architecture and the leader-based consistency maintenance mechanism. Section 4 describes a default leader election algorithm in Kubernetes, and the leader concentration problem. Section 5 presents the proposed balanced leader distribution algorithm. Section 6 and Section 7 present the performance evaluation and discussion, respectively. Finally, Section 8 presents the conclusions.

## 2. Related Work

Recently, there have been several studies based on Kubernetes. The paper [8] proposed a prediction model to improve the performance of auto-scaling in Kubernetes. This model combines empirical modal decomposition with an autoregressive integrated moving average model to predict the load of the pod. The goal is to expand the capacity of the application before the peak load by adjusting the number of pods in advance according to the prediction result. In [9], the authors investigate the horizontal pod autoscaler (HPA), which is one of the most important features in Kubernetes. They conduct various experiments to deeply analyze HPA based on several metrics, such as Kubernetes resource metrics (e.g., CPU and memory usage) and Prometheus custom metrics (e.g., the average arrival rate of HTTP requests) [10]. Based on the analysis, researchers and developers can gain a deep insight on optimizing the performance of HPA in Kubernetes. In [11], a monitoring platform was presented for dynamic resource provisioning based on Kubernetes. It collects the system resource use (CPU, RAM) and application quality-of-service metrics (response time) by using several open-source applications, such as Heapster and Apache JMeter [12]. Based on these data, it automatically analyzes and scales the application according to the resource provisioning strategy. The paper [13] presented a Reference Net-based performance and management model for Kubernetes. The goal is to identify the effects of the different interference sources (e.g., CPU usage and network usage) on the applications; therefore, the developer can consider such interference sources and improve the application’s performance. In [14], a component in Kubernetes was used to build a protocol, named DORADO (orDering OveR shAreD memOry), which coordinates requests in Kubernetes. A leader is elected from among the replicas of an application. To coordinate the requests accessing the application, only the leader has the authority to define orders for handling requests, and all the replicas must execute the requests following this order. In [15], the authors proposed a load balancer for Kubernetes. The proposed load balancer can consider the running status of servers and applications (e.g., CPU and network status) to distribute requests. The users can configure the load balancing rules based on several metrics, including CPU, memory usage, and network IO. Subsequently, the server running status is collected, and the real-time load of each server is calculated to find a back-end server that can forward the requests. The paper [16] proposed a solution that allows for automatically redirecting client requests to healthy pods. A state controller was implemented to integrate with Kubernetes, and it can determine the status of a pod and assign the “active” or “standby” label to the pod. The client requests are redirected to the pods that have the “active” label, and the messages containing the state data are replicated to the standby pods. The paper [17,18] proposed a network-aware scheduling approach that is extended from the default scheduling mechanism in Kubernetes. This approach is used to deploy container-based applications in a smart city. In the Fog computing environment, the paper [19] presented a framework that is based on Kubernetes. It collects the network traffic status to provide elastic resource provisioning of the container-based application among geographically distributed Fog nodes in real time. Additionally, a few experiments were conducted to evaluate the efficiency of Kubernetes on NFV management and orchestration [20] or on deploying microservice-based applications [21].

Regarding the consensus problem in distributed systems, many studies have been conducted over several decades. Some well-known algorithms have been applied to ensure a consensus of data among replicas in distributed systems. Paxos [22], proposed by Lamport, is one of the most famous distributed consensus algorithms. One or more proposed values are proposed to Paxos, and the consensus is achieved when a majority of the replicas accept one of the proposed values. Raft [23] is a consensus algorithm that applies specific techniques that make it more understandable than Paxos. It separates the consensus problem into relatively independent subproblems, such as leader election and log replication. In OpenDaylight (ODL), which is an open source project for Software Defined Networking (SDN) controller, the datastore is distributed into shards; and these shards can be located in any node of the cluster [24]. The Raft algorithm was implemented to maintain the consistency in these distributed datastores. Paper [25] integrated Raft consensus algorithm with Kubernetes. They present evaluations of the Raft algorithm running on the physical machine and on containers managed by Kubernetes. The results showed that the throughput when executing the Raft algorithm on Kubernetes approximately was 17.4% lower than that when running directly on a physical machine; however, it is acceptable because of the many powerful features offered by Kubernetes. Paper [26] presents a solution for replica stateful containers management in Kubernetes. A coordination layer that uses Raft as a consensus algorithm was implemented. A leader replica was determined from among the replicas of an application, and the write operations were performed by only the leader replica. However, the proposed approach is complicated because it requires developers to integrate the Raft algorithm into Kubernetes and implement a firewall to redirect the requests to the leader replica.

To simplify the use of the leader election process, a leader election algorithm was implemented by leveraging existing information and components in Kubernetes [6]. However, as demonstrated in [7], this leader election algorithm does not consider the leader concentration problem in a specific node of the cluster, which results in the bottleneck problem and decreases the system performance. In this paper, a new leader election algorithm is proposed to solve this problem by attempting to balance the number of leaders among nodes in the Kubernetes cluster.

## 3. Overview of Kubernetes

This section presents an overview of the Kubernetes architecture and a leader-based consistency maintenance mechanism for stateful applications in the Kubernetes cluster.

### 3.1. Kubernetes Architecture

**Figure 1:**
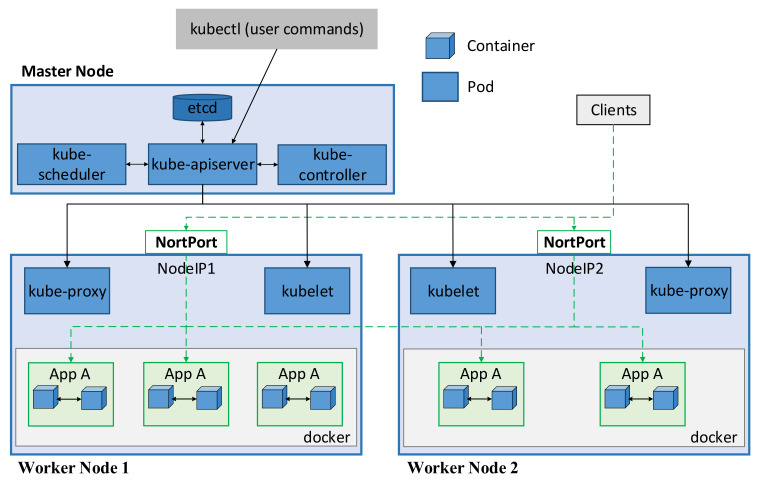
Kubernetes architecture.

Kubernetes is a well-known open-source platform for automating the deployment, scaling, and management of container-based applications. A pod, which is the smallest unit in Kubernetes, represents a single instance of an application. Each pod contains a set of one or more containers, and these containers are tightly coupled, use the same IP and data storage. The architecture of Kubernetes is shown in Figure 1. A Kubernetes cluster usually has one or more master nodes and several worker nodes. The master node is the control plane that is responsible for managing and controlling the cluster. It contains four main components: *etcd*, *kube-apiserver*, *kube-scheduler*, and *kube-controller* [4]. The *etcd* is a datastore that is used to store the configuration and the state of the cluster. The *kube-apiserver* is the front end of the Kubernetes control plane. In other words, the user or management request needs to communicate with the *kube-apiserver* to interact with the Kubernetes cluster. The *kube-scheduler* watches unscheduled pods and assigns them to a node to run on based on multiple factors, such as resource constraints, affinity, and anti-affinity rules. The *kube-controller* continuously watches the state of the cluster to maintain the desired state. For example, it ensures the correct number of running replicas of an application according to the desired configuration.

The pods are scheduled and orchestrated to run on the worker nodes that consist of three main components: *kubelet*, *kube-proxy*, and *container runtime* [4]. *Kubelet* ensures that the pods are running and healthy (e.g., by restarting failed pods). It reports the status of pods and node to the api-server and receives commands from the control plane. *Kube-proxy* is responsible for maintaining the network rules, which allow communication with the pods from inside and outside of the cluster. *Container runtime* (e.g., Docker [27] or containerd [28]) pulls the container image from a container registry and deploys the container based on that image. In Kubernetes, a pod can be created and destroyed frequently, and its IP address is updated after a restart; therefore, it is difficult to access an application using the pod’s IP address. Kubernetes provides a Service that is an abstract layer enabling network access to a set of pods. The pods are selected based on their label, and all pods belonging to a Service have the same label. The Service is assigned an unchanging IP address (ClusterIP), and the requests accessing the Service are load-balanced among the pods. The load balancing policy depends on the proxy mode of *kube-proxy*. By default, the *userspace* mode uses a round-robin algorithm to select the pods, whereas the *iptable* mode selects pods randomly. The *IP Virtual Server* can load balance traffic to the pods in several ways, such as destination hashing, source hashing, and round-robin methods. The ClusterIP is reachable only from within the cluster. To expose the application outside the cluster, the NodePort and LoadBalancer Service can be used. The NodePort Service exposes the application on the node’s IP address at a static port (NodePort). As shown in Figure 1, clients from outside the cluster can access to the NodePort Service by using the NodeIP:NodePort address. The traffic accessing the NodePort Service is then forwarded to a backend pod according to the configuration in *kube-proxy*. The LoadBalancer Service exposes the application externally using a cloud provider that provides a public IP address, and the load balancing policy depends on the cloud provider implementation.

### 3.2. Leader-Based Consistency Maintenance Mechanism

Stateful applications are services that require saving data to persistent data storage, such as a database or key-value store, for use by servers, clients, and other applications [29]. The pods in Kubernetes are ephemeral in nature and do not persist data, so the data in a pod is lost once it is destroyed or restarted. To support persistent data storage, Kubernetes provides a PersistentVolume (PV) and a PersistentVolumeClaim (PVC) object. A PV is a persistent storage that has an independent lifecycle with the pod. A PVC defines several criteria (e.g., capacity and access mode) to choose the persistent storage, so it is used to claim a persistent storage that satisfies the criteria. Therefore, each pod replica of the stateful application can create its own persistent data storage by using the PVC. This PVC binds the pod to a PV that satisfies the criteria defined in the PVC.

**Figure 2:**
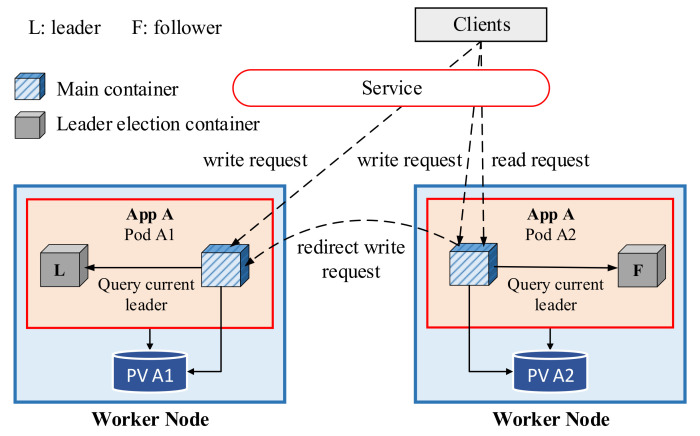
Leader-based consistency model [7].

In Kubernetes, one application can have multiple replicas to provide high availability and performance. For example, throughput and latency can be improved by using the load balancing feature in Kubernetes, which distributes the incoming requests among replicas of the application. Because each replica of the stateful application has its own data storage, deploying a set of replicas for the stateful application requires an approach to handle the inconsistency problem among these distributed databases. To handle this problem in the Kubernetes cluster, the paper [7] introduced a leader-based consistency mechanism, as shown in Figure 2. In this mechanism, a replica is elected as a leader, and the other replicas run as the followers. Read operations that clients require to read data from the storage is handled by both the leader and follower. However, only the leader is responsible for handling write operations that clients write the data into the storage. Thus, if a request for a write operation comes to a follower, it must be redirected and handled by the leader.

To determine the leader among the pod replicas, each pod consists of two containers: a main container and a leader election container. The main container is responsible for handling incoming requests from clients, whereas the leader election container is responsible for the leader election process among replicas of the application. The leader election container provides a simple web server that returns the name of the current leader; thus, the main container can easily determine its role (leader or follower) by querying this web server in the leader election container.

## 4. Default Leader Election Algorithm in Kubernetes

This section presents the default leader election algorithm in Kubernetes and discusses the leader concentration problem of the default algorithm.

### 4.1. Default Leader Election Algorithm in Kubernetes

To use the leader-based consistency maintenance mechanism in a distributed system, an approach to elect a leader among the replicas is essential. Implementing leader election often requires deploying either algorithms or software such as Raft [23], Zookeeper [30], or Consul [31]. However, to avoid high implementation costs and facilitate the use of leader election in the Kubernetes cluster, a simple leader election algorithm was implemented by leveraging existing components in Kubernetes [6].

**Figure 3:**
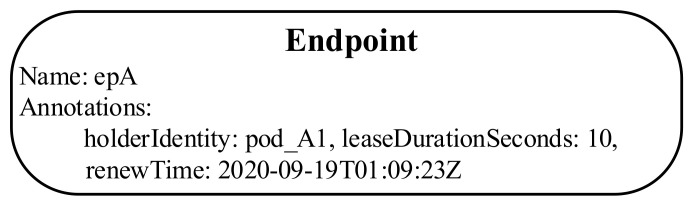
Example of leader record in the EP.

Typically, in a leader election algorithm, a set of candidates compete to become a leader in several ways. For example, the first one who successfully declares itself as a leader or the candidate who receives a majority of votes from other candidates can become a leader. Once the leader election process is completed, the leader continuously sends “heartbeats” to retain the leadership. If the current leader fails for some reason, the other candidates can be aware of that status and start a new election process to become the leader. The leader election algorithm in Kubernetes uses an annotation in the Endpoint object (EP) to hold a leader record. An example of the leader record in the EP is shown in Figure 3. The leader record includes the name of the leader (*holderIdentity*), the time when the leader renews the leader record in EP (*renewTime*), and the timeout duration (*leaseDurationSeconds*) that the follower has to wait to acquire the leadership.

**Figure 4:**
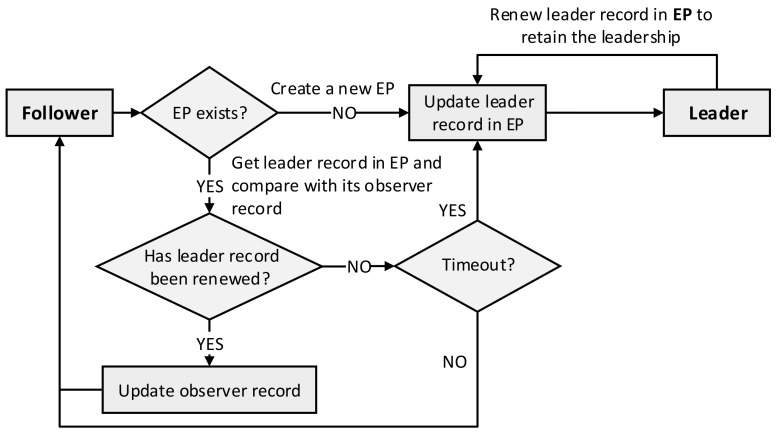
Procedure of the default leader election algorithm.

The procedure of the default leader election algorithm is presented in Figure 4. Once a replica starts, it runs as a follower and periodically checks the leader record in the EP to try to acquire the leadership. Please note that each replica maintains an observer record that contains the leader record copied from the EP and the observer time when the observer record was updated. First, the follower checks for the existence of an EP; if an EP has not yet been created, it creates a new EP and updates the leader record in that EP to become a new leader. If the EP did exist, the follower obtains the leader record from the EP and compares it with its own observer record to determine whether the leader record was renewed or not. If the leader record was renewed (the leader record in the EP differs from the leader record in the observer record), it updates the observer record and remains in the follower state. Otherwise, it checks the timeout by calculating the total elapsed time from the latest observation (when the observer record was updated) to the current time. If this value exceeds the predetermined timeout, the candidate updates the leader record in the EP to become a new leader. Otherwise, it remains in the follower state and periodically tries to acquire the leadership by checking the EP. The leader also has to periodically renew the leader record by updating the *renewTime* in the EP to retain its leadership.

### 4.2. Leader Concentration Problem

**Figure 5:**
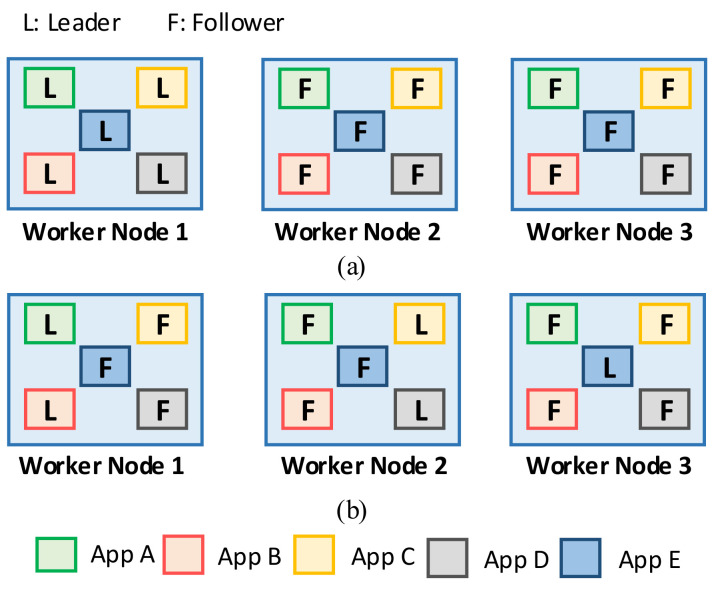
Example of leader distribution: (**a**) concentrated leaders, (**b**) balanced leaders.

**Figure 6:**
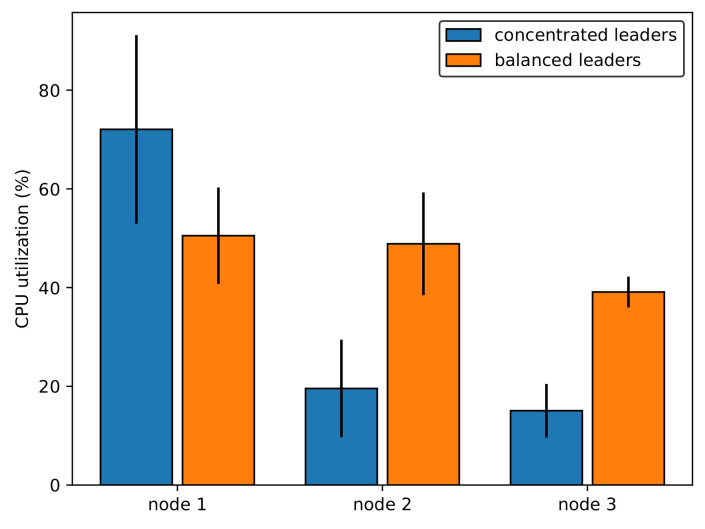
CPU use on each node when clients send write requests.

We consider a scenario in which several stateful applications are deployed in the Kubernetes cluster as a Fog computing infrastructure. Each application has multiple pod replicas, and the application employs the leader-based mechanism for maintaining consistency among data storage of the replicas. It is obvious that the workload of the leader is higher than that of the followers because all requests for the write operation are handled only by the leader. Therefore, if the leaders of these applications are concentrated on a specific node, it may lead to a workload imbalance problem among worker nodes–one node with many leaders has a heavier workload than other nodes do. Eventually, it can cause a bottleneck in the cluster, which results in a significant decrease in the system performance, as already discussed by [7,24]. An example of the leader distribution is shown in Figure 5. There are three worker nodes, and five applications are deployed in this cluster. Figure 5a presents a concentrated leader distribution (concentrated leaders) with five leaders in node 1, while Figure 5b presents a balanced leader distribution (balanced leaders) with 2, 2, and 1 leaders in node 1, node 2, and node 3, respectively. To prove the workload imbalance problem, four clients simultaneously send write requests to each application over a period of time. Figure 6 shows the average CPU use and standard deviation of each worker node in concentrated leaders and balanced leaders. In the case of concentrated leaders, the average of CPU use in node 1 is approximately 70%, while that in node 2 and node 3 is only approximately 20%. In the case of balanced leaders, we can observe a balanced CPU use among nodes, which is approximately 50% in both node 1 and 2 and approximately 40% in node 3. Therefore, it is clear that the workload imbalance problem can occur when five leaders are concentrated in a specific node. This hinders the ability to fully exploit the computational and networking resources of the distributed system. Meanwhile, the workload can be balanced among worker nodes in the cluster when the leader distribution is balanced.

Moreover, it is important to note that although the default leader election algorithm in Kubernetes can facilitate the use of leader election in the Kubernetes cluster, it does not consider where the leader is running. Consequently, it may lead to a leader concentration problem on a specific node, resulting in a significant decrease in the system performance.

## 5. Balanced Leader Distribution Algorithm

**Figure 7:**
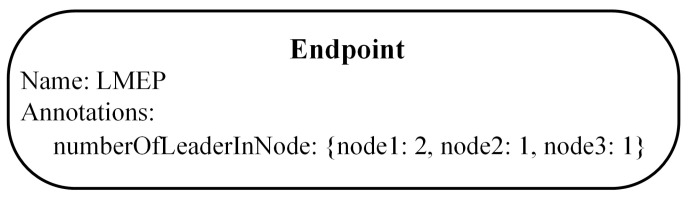
Example of leader information in the LMEP.

**Figure 8:**
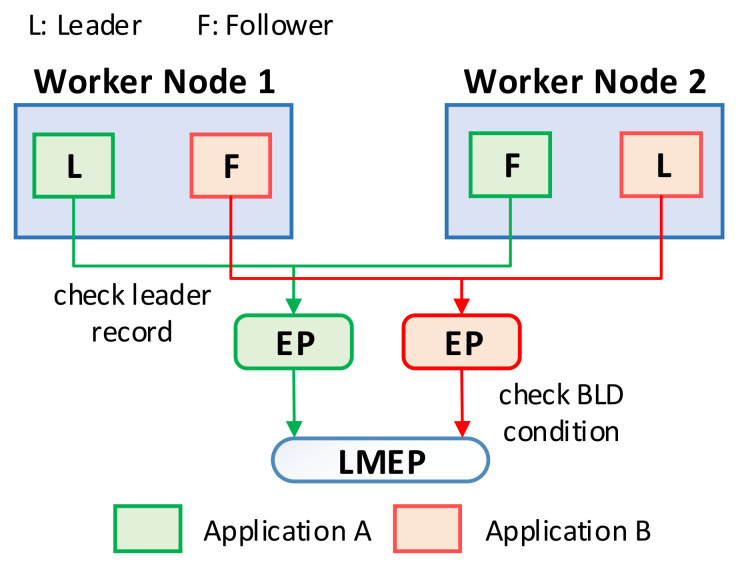
Overview procedure of the BLD algorithm.

In this section, we present the BLD algorithm that overcomes the weakness of the default leader election algorithm in Kubernetes. The proposed algorithm considers the number of leaders in the nodes to achieve a balanced leader distribution among nodes in the Kubernetes cluster. To store the information of the number of leaders on each node, we newly define an Endpoint object named Leader Management EP (LMEP), as shown in Figure 7. The number of leaders on the node is updated by the leader of the application. Once a new leader is determined, it realizes the node where it is running by using Kubernetes API and updates the leader information in LMEP. Using this information, we can calculate the current total number of leaders in the cluster (*L*${}_{cluster}$). The number of worker nodes (*N*) can also be retrieved by using Kubernetes API. Let us assume that the maximum number of leaders on each node is denoted as *M*; the value of *M* can be calculated as *M* = (*L*${}_{cluster}$ + 1)/*N*, to balance the number of leaders on each node. The balanced leader distribution condition is satisfied if the number of leaders in the node where the candidate is running is smaller than or equal to *M*. The overview of the algorithm is shown in Figure 8. First, the replica frequently checks the leader record in the EP to try to become a leader (if it is a follower) or to renew the leader record to retain the leadership (if it is a leader). After satisfying the original conditions of the default algorithm, the follower obtains the leader information of the cluster from the LMEP and checks the BLD condition to investigate the status of the leader distribution. It becomes a leader if the BLD condition is satisfied.

**Figure 9:**
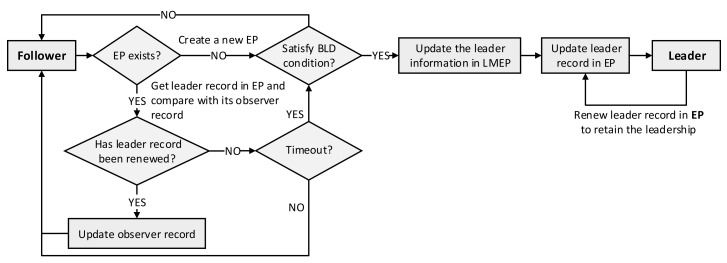
Detailed procedure of the BLD algorithm.

The detailed procedure of the BLD algorithm is depicted in Figure 9. The replica pods start as followers, and they race to become a leader by trying to be the first one who successfully declares itself as a leader in the EP. As in the default algorithm, the candidate checks the existence of the EP. If the EP has not yet been created, it creates a new one. Then, the BLD condition is checked. If it satisfies the condition, it updates the leader information in the LMEP and EP to become a leader. Otherwise, if the EP already existed, steps similar to the default algorithm are performed—it checks the leader record in EP, updates the observer record, and checks whether the timeout duration is over. If the timeout has not expired, it remains as a follower. Otherwise, the candidate checks the BLD condition. If it satisfies the BLD condition, it updates the information in the LMEP and EP to become a new leader. If it does not satisfy the BLD condition, it remains as a follower and periodically tries to acquire leadership by repeating the aforementioned procedure. Similarly, the leader periodically renews the leader record in the EP to retain its leadership.

## 6. Performance Evaluation

In this section, we describe our experimental setup. Then, we compare the evaluation results of the leader distribution and leader election latency between the default algorithm and BLD algorithm. Finally, the effects of the leader distribution is analyzed in terms of throughput.

To evaluate the correctness and effectiveness of the BLD algorithm, we set up a Kubernetes cluster that contains one master node and three worker nodes, using Kubernetes version 1.14.10 and Docker version 18.09.6. The master node has 4 GB of RAM and four CPU cores, and the three worker nodes have 3 GB of RAM and four CPU cores. Several stateful applications are deployed in the cluster. The Hey program [32] is used to create and send requests to the applications.

### 6.1. Leader Distribution and Leader Election Latency

**Figure 10:**
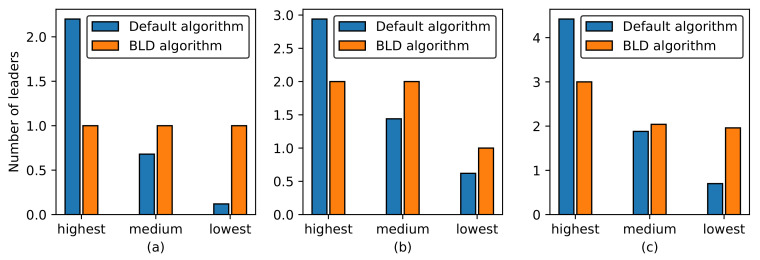
Leader distribution among nodes in the cluster: (**a**) 3 applications; (**b**) 5 applications; (**c**) 7 applications.

To evaluate the leader distribution and the leader election latency, we deploy a different number of stateful applications that use the leader-based mechanism to maintain consistency among replicas of the application. Each application is set to have five replicas, and the experiment is repeated 100 times. Figure 10 shows a comparison of the leader distribution among the worker nodes between using the default algorithm and using the BLD algorithm. The number of leaders in each node is sorted in a descending order; therefore, highest, medium, and lowest indicate the highest, medium, and lowest number of leaders concentrated in one node. For three applications, the leader distribution is 2.2:0.68:0.12 using the default algorithm, whereas it is 1:1:1 using the BLD algorithm. For five and seven applications, the leader distribution in the default algorithm is unbalanced among worker nodes, with 2.94:1.44:0.62 and 4.42:1.88:0.7, respectively. The leader distribution in the BLD algorithm is balanced among worker nodes, with 2:2:1 and 3:2.04:1.96 for five and seven applications, respectively. The standard deviation, minimum and maximum number of leaders in a node are presented in Table 1. We can see that a high number of leaders can be concentrated in a specific node with the default algorithm. For example, all leaders of the applications may be concentrated in one node in case the number of applications are three and five. Therefore, it is clear that the leader distribution among nodes is balanced when the BLD algorithm is applied, whereas it is unpredictable when the default algorithm is used.

**Figure 11:**
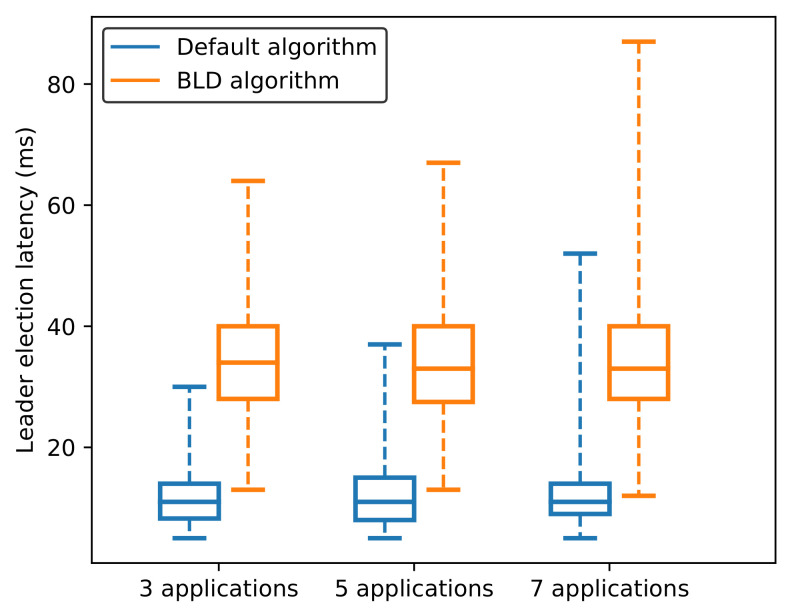
Leader election latency.

Figure 11 shows an analysis of the latency of the leader election process, measured from when a new election process starts until a candidate becomes a leader. The average leader election latency in the case of the default algorithm is approximately 12 ms for three, five, and seven applications. This is because the default algorithm does not consider where the leader is; therefore, the first replica that starts the election process is highly possible to become a leader. The average latency for the leader election process in the case of the BLD algorithm is slightly higher than in the default algorithm, approximately 34 ms for three, five, and seven applications. Besides, the variation and the maximum value of the leader election latency in the case of the BLD algorithm are also higher than that in the default algorithm. This is because the BLD algorithm requires additional rounds of leader election in case the BLD condition is not satisfied. Table 2 presents the mean, standard deviation of the results, and maximum and minimum obtained values for the leader election process. Clearly, they are higher than the values obtained using the default algorithm; however, it can be considered to be a trade-off to improve throughput, which is shown in the next subsection.

### 6.2. Effect of Leader Distribution in Kubernetes Cluster

**Figure 12:**
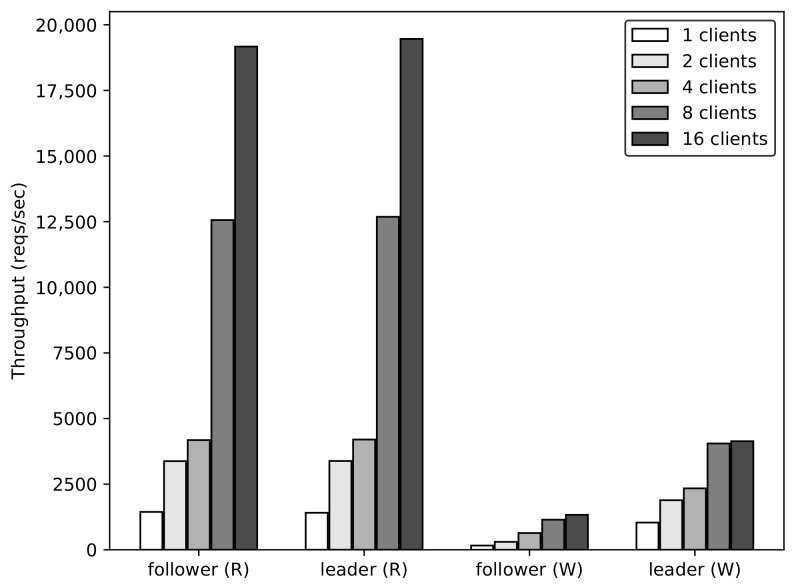
Throughput of one application according to replica’s role and read/write operation.

Here, we analyze the throughput according to the replica’s role and the leader distribution. The number of concurrent clients accessing each application is increased from 1 to 16. The requests are sent to the applications for 60 s. To evaluate the throughput of read and write operation based on the replica’s role, one application with five replicas is deployed. The requests are sent directly to the follower or the leader of the application. The evaluation results are shown in Figure 12. For the read operation, the leader and the follower have a similar trend with increasing concurrent requests, because the read operation can be handled immediately by the leader or follower. The throughput for the write operation handled by a follower is significantly lower than that handled by a leader. For example, the throughput for the write operation in the case of the follower is 161.55 and 1330.7 reqs/s with 1 and 16 concurrent clients, respectively; whereas, in the case of the leader, it is 1037.13 and 4136.76 reqs/s, respectively. This is because the write requests are handled only by the leader; if a request comes to a follower, it will be redirected to the leader. Please note that the write operation requires more computational resources and time to handle the requests than the read operation does; this is why the throughput of the write operation is considerably lower than that of the read operation. In the case of one application, we can conclude that the throughput of the write operation can be significantly improved if the requests are handled directly by the leader, and the write operation takes more time and resources to handle than the read operation does.

**Figure 13:**
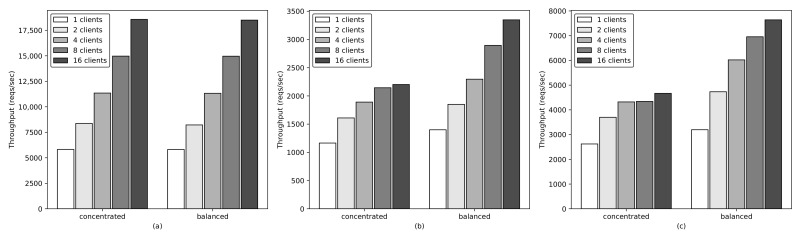
Cumulative throughput of multiple applications according to leader distribution: (**a**) Read operation. (**b**) Write operation. (**c**) Smart write operation.

To analyze the importance of balanced leader distribution, we evaluate the throughput between two leader distribution scenarios: “concentrated leaders” and “balanced leaders”, which are representation results for the default leader election algorithm and the BLD algorithm, respectively. Five applications, each of which contains five replicas, are deployed in the cluster. In case of “concentrated leaders”, all the leaders of these applications are assumed to be concentrated in one specific node. In case of “balanced leaders”, the number of leaders in each node is balanced, such that three worker nodes have 2:2:1 leaders for five applications. In this experiment, we use the NodePort service in Kubernetes to expose the service of the application to the outside cluster. The requests accessing the application through the NodePort are distributed among replicas of the application using the *iptables* proxy mode. Figure 13 shows the cumulative throughput of five applications according to the leader distribution scenarios and the request operations (read, write, and smart write operation). For the read and write operation, the requests access the application through a NodePort service, and they can be redirected to either a follower or a leader. The smart write operation is defined as forwarding the write requests directly to the leader of the application to avoid the overhead of forwarding requests to the leader in the leader-based consistency maintenance mechanism. Figure 13a shows that the throughput for the read operation in both “concentrated leaders” and “balanced leaders” has a similar trend because requests are handled immediately by any replica. Meanwhile, the throughput for the write operation in the case of “balanced leaders” is significantly higher than that in the case of “concentrated leaders”, as shown in Figure 13b. The throughput obtained with 1 client in the case of “balanced leaders” is approximately 20.16% higher than that in the case of “concentrated leaders”. It tends to become worse as the number of concurrent clients increases. For example, the throughput in the case of “balanced leaders” is approximately 35%, 52.03% higher than that in the case of “concentrated leaders” with 8 and 16 concurrent clients, respectively. In the case of “concentrated leaders”, because all the write requests are handled at one specific node and that node reached its maximum capacity. By contrast, in case of “ balanced leaders”, the write requests are distributed throughout the nodes in the cluster; therefore, the throughput keeps increasing as the number of concurrent requests increases. Figure 13c shows the throughput for the smart write operation; the “balanced leaders” case shows a significant improvement over “concentrated leaders” in terms of throughput. For example, the throughput in the “balanced leaders” case is approximately 21.93% higher than that in the “concentrated leaders” case with 1 concurrent client, and it is approximately 63.7% with 16 concurrent clients. Notably, the write request is forwarded directly to the leader in the smart write operation, whereas in the normal write operation, it is randomly forwarded to the replicas regardless of their roles. Therefore, the throughput obtained in the smart write operation is significantly higher than that obtained in the normal write operation. In the case of “balanced leaders”, the throughput obtained with 1 and 16 concurrent requests in the normal write operation is 1399.76 and 3347.77 reqs/s, respectively; whereas it is 3196.41 and 7640.08 reqs/s, respectively, in the smart write operation. Overall, we can conclude that balancing the leaders of multiple stateful applications among nodes significantly improves the performance, and it can be further enhanced by implementing a smart network service that can forward requests to an appropriate replica according to the replica’s role.

## 7. Discussion

Throughout the performance evaluations, we have proved that the proposed BLD algorithm evenly distributes multiple leaders to nodes in the cluster and enhances the throughput of the cluster by balancing the workload of nodes. However, it is worth discussing the limitation of the BLD algorithm. As we have discussed in Section 6.1, the BLD algorithm causes a relatively high leader election latency compared to the default algorithm due to the design of the BLD condition check. The absence of a leader can lead to temporal service interruption. Moreover, although the leader election latency of the BLD algorithm in our experimental environment is not significantly high, there is a possibility that it may increase as the number of replicas and applications increase. Since the absence of a leader may lead to temporal service interruption, in the future works, we will investigate the effect of the leader election latency in large-scale infrastructures to improve both throughput and availability of the service.

It is also interesting to note that the throughput can be improved significantly in case the smart write operation is applied. Hence, implementing a network service in Kubernetes that is aware of the role of a replica and can forward requests to an appropriate replica according to its role is worth considering in future works.

## 8. Conclusions

In this paper, we described the Kubernetes architecture and the leader-based mechanism for maintaining consistent data storage among replicas of a stateful application in the Kubernetes cluster. Because the leader concentration problem can cause unbalanced resource usage among nodes, the full exploitation of the computational resources of the cluster is hindered. Therefore, we proposed a leader election algorithm that not only facilitates the use of leader election in Kubernetes but also evenly distributes the leaders throughout all the nodes in the cluster. The evaluation results showed that the proposed BLD algorithm can effectively balance the number of leaders among all nodes in the cluster. The effectiveness of the BLD algorithm was proved through a performance evaluation with multiple applications, demonstrating that the throughput can be significantly improved by distributing the number of leaders evenly throughout the nodes. There have been more and more systems using the leader-based mechanism, and we expect that the idea of a balanced leader distribution throughout the nodes is widely applied to leader election algorithm design, to maximize the performance of the cluster.

## Figures and Tables

**Table 1:** Statistics of leader distribution.

Algorithm	Default			BLD		
**Number of Applications**	**3**	**5**	**7**	**3**	**5**	**7**
Std. dev.	1.01	1.2	1.79	0	0.47	0.5
Minimum value	0	0	0	1	1	2
Maximum value	3	5	6	1	2	3

**Table 2:** Statistics of leader election latency.

Algorithm	Default			BLD		
**Number of Applications**	**3**	**5**	**7**	**3**	**5**	**7**
Mean value (ms)	11.91	12.44	11.92	34.46	33.41	33.77
Std. dev. (ms)	4.65	5.71	5	10.51	10.32	10.93
Minimum value (ms)	5	5	5	13	13	12
Maximum value (ms)	30	37	52	64	67	87
